# NMR‐Based Analysis of Nanobodies to SARS‐CoV‐2 Nsp9 Reveals a Possible Antiviral Strategy Against COVID‐19

**DOI:** 10.1002/adbi.202101113

**Published:** 2021-10-27

**Authors:** Gennaro Esposito, Yamanappa Hunashal, Mathias Percipalle, Tomas Venit, Mame Massar Dieng, Federico Fogolari, Gholamreza Hassanzadeh, Fabio Piano, Kristin C. Gunsalus, Youssef Idaghdour, Piergiorgio Percipalle

**Affiliations:** ^1^ Chemistry Program, Science Division New York University Abu Dhabi Abu Dhabi 129188 United Arab Emirates; ^2^ Istituto Nazionale Biostrutture e Biosistemi Roma 00136 Italy; ^3^ Department of Chemistry and Magnetic Resonance Center University of Florence Florence 50019 Italy; ^4^ Biology Program, Science Division New York University Abu Dhabi Abu Dhabi 129188 United Arab Emirates; ^5^ Dipartimento di Scienze Matematiche, Informatiche, e Fisiche Udine University Udine 33100 Italy; ^6^ VIB Nanobody Core Vrije Universiteit Brussel Brussels 1050 Belgium; ^7^ Public Health Research Center New York University Abu Dhabi Abu Dhabi 129188 United Arab Emirates; ^8^ Center for Genomics and Systems Biology New York University Abu Dhabi (NYUAD) P.O. Box 129188 Abu Dhabi United Arab Emirates; ^9^ Department of Biology Center for Genomics and Systems Biology New York University New York NY 10003 USA; ^10^ Department of Molecular Bioscience The Wenner Gren Institute Stockholm University Stockholm S‐106 91 Sweden

**Keywords:** COVID‐19, nanobodies, NMR spectroscopy, Nsp9, SARS‐CoV‐2

## Abstract

Following the entry into the host cell, SARS‐CoV‐2 replication is mediated by the replication transcription complex (RTC) assembled through a number of nonstructural proteins (Nsps). A monomeric form of Nsp9 is particularly important for RTC assembly and function. In the present study, 136 unique nanobodies targeting Nsp9 are generated. Several nanobodies belonging to different B‐cell lineages are expressed, purified, and characterized. Results from immunoassays applied to purified Nsp9 and neat saliva from coronavirus disease (COVID‐19) patients show that these nanobodies effectively and specifically recognize both recombinant and endogenous Nsp9. Nuclear magnetic resonance analyses supported by molecular dynamics reveal a composite Nsp9 oligomerization pattern and demonstrate that both nanobodies stabilize the tetrameric form of wild‐type Nsp9 also identifying the epitopes on the tetrameric assembly. These results can have important implications in the potential use of these nanobodies to combat viral replication.

## Graphical abstract

New nanobodies targeting Nsp9, a nonstructural protein required for Sars‐CoV‐2 replication, are generated. These nanobodies specifically recognize Nsp9 in saliva samples from COVID‐19 patients and stabilize a tetrameric form of Nsp9, not compatible with its role in facilitating viral replication. These results potentially identify novel diagnostic and therapeutic antiviral strategies to combat Sar‐CoV‐2 and, therefore, COVID‐19.

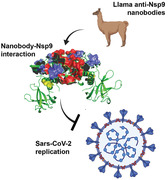

## 1 Introduction

The ongoing coronavirus disease (COVID‐19) pandemic is caused by severe acute respiratory syndrome coronavirus 2 (SARS‐CoV‐2), a positive‐sense single‐stranded RNA (ssRNA) virus. The development of new antiviral drugs would, therefore, facilitate and potentiate treatment, to eventually eradicate infection and emergence of escape variants of the virus or other coronaviruses.

Nanobodies are the variable domains of heavy chain antibodies (HCAbs), a component of the antibody repertoire of camelids. Nanobodies bind their antigens also when used as single domains devoid of the constant HCAbs frame.^[^ ^1^ ^]^ Several nanobodies have been generated against the surface‐exposed portion of Spike with the aim of blocking viral entry in the host cell.^[^ ^2^, ^3^ ^]^ Here, we sought to identify other potential targets for development of nanobodies that could have potential use in diagnostics and, possibly, treatment. We targeted the multi‐subunit replication transcription complex (RTC), whose subunits are encoded by two large open reading frames (ORFs).^[^ ^4^ ^]^ Recently, structural snapshots of the SARS‐CoV‐2 RTC have been reported at atomic resolution. The complex is assembled by Nsp7‐(Nsp8)_2_‐Nsp12‐(Nsp13)_2_‐RNA and a single RNA‐binding protein, Nsp9, which is necessary for RTC function.^[^ ^4^ ^]^ Although Nsp9 has a strong tendency to oligomerize,^[^ ^5^, ^6^, ^7^ ^]^ within the RTC it appears to be in a monomeric state.^[^ ^8^ ^]^ As a monomer, Nsp9 interacts with the Nsp12 (RdRp) NiRAN catalytic domain, which has nucleoside monophosphate (NMP) transferase activity, leading to the covalent attachment of a nucleoside monophosphate to the evolutionarily conserved Nsp9 amino terminus, a critical step in the initiation of viral replication.^[^ ^9^ ^]^

## 2 Results and Discussion

**Figure 1:**
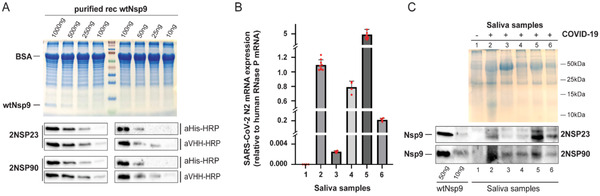
Llama derived nanobodies specifically cross‐react with Nsp9 in COVID‐19 saliva samples. A) Decreasing amount of purified recombinantly expressed Nsp9 (purified rec wtNsp9) preincubated with BSA were separated by SDS PAGE, immunostained with nanobodies 2NSP23 and 2NSP90. Detection was with HRP‐conjugated secondary antibodies to 6xHis tag (aHis‐HRP) or to the VHH domain (aVHH‐HRP). B) RTqPCR analysis of Sars‐Cov‐2 N2 mRNA levels as proxy for viral load. mRNA levels were normalized against human RNase P mRNA. Each red dot represents a single measurement value. Each column represents a mean value from at least 4 independent measurements (*n* ≥ 4). Error bar represents the standard deviation from the mean value C) 15 µg of saliva protein samples from COVID‐19 negative and positive individuals were loaded together with 50 and 10 ng of purified NSP9 which served as a positive control. Top panel, SDS PAGE, bottom panel, corresponding immunoblots with nanobodies 2NSP23 and 2NSP90.

To select for nanobodies against Nsp9, a llama was immunized with a recombinant SARS‐CoV‐2 Nsp9 protein carrying three mutations, C14S, C23S, and C73S (triSer‐Nsp9), to prevent oxidation of free Cys SH groups that could elicit heterogeneity in the immune response. Molecular dynamics simulations of wild‐type and mutant Nsp9 show high similarity (Figure S1, Supporting Information). After the last immunization, anticoagulated blood was collected to prepare peripheral blood lymphocytes (PBLs) and library generation to screen for the presence of antigen‐specific nanobodies. The details of the procedure are described in the Supporting Information. Overall, enzyme‐linked immunosorbent assay (ELISA) tests performed on immobilized triSer‐Nsp9 identified 136 different nanobodies, belonging to 40 different CDR3 groups (B‐cell lineages) (Table S1, Supporting Information). We next selected eight Nsp9‐specific nanobody genes from 8 different CDR3 groups. These genes were cloned, expressed in *Escherichia coli* WK6 and purified by IMAC and size exclusion chromatography (Figure S2, Supporting Information). Sequences, annotations and analytical characterizations are given in Figure S3 and Table S2 in the Supporting Information. For further characterization, we selected nanobodies 2NSP23 and 2NSP90 and tested for binding to wild‐type Nsp9 on immunoblots (**Figure** **1**a). After incubation with the membrane, nanobodies were detected with secondary antibodies recognizing either the llama VHH domain or His6 tag fused to both 2NSP23 and 2NSP90 nanobodies (Figure 1A). Results from immunoblotting show that Nsp9 was specifically recognized by 2NSP23 and 2NSP90 at antigen concentrations as low as 25 ng per loading (1.25ng µL^−1^).

We next examined if nanobodies 2NSP90 and 2NSP23 specifically bind Nsp9 in biological samples. Saliva from individuals infected with COVID‐19 was collected in sterile containers, per a recent study demonstrating that saliva can be used for SARS‐CoV‐2 detection by RT‐PCR.^[^ ^10^ ^]^ To confirm the presence of SARS‐CoV‐2 in the saliva samples, we monitored expression levels of mRNA encoding the viral N2 protein using real time qPCR. Significantly high N2 mRNA levels, normalized to expression of human RNase P mRNA, were observed in saliva from the five COVID‐19 patients but not in a saliva sample from a healthy donor used as negative control (Figure 1B). COVID‐19 positive samples exhibited different N2 mRNA levels, compatible with different viral loads (Figure 1B). To test if nanobodies 2NSP90 and 2NSP23 can detect Nsp9 in neat saliva from COVID‐19 patients, we extracted proteins by diluting saliva samples in SDS loading buffer. Following heat denaturation, samples were electrophoresed under denaturing conditions and transferred on a membrane. For the immunoassays, membranes were separately incubated with nanobodies 2NSP90 and 2NSP23 followed by tagged secondary anti‐VHH antibodies for visualization (Figure 1C). We detected a specific signal across all COVID‐19 patients’ samples with both nanobodies. Neither 2NSP90 nor 2NSP23 exhibited a positive signal in the sample from the healthy donor, indicating a degree of specificity toward their antigen. The differences in the amounts of detected Nsp9 mirror differences in viral loads measured by real time qPCR. A comparison with purified Nsp9, loaded as control (50 and 10 ng), suggests that 2NSP90 and 2NSP23 can detect as little as 10 ng of Nsp9 protein in saliva.

**Figure 2:**
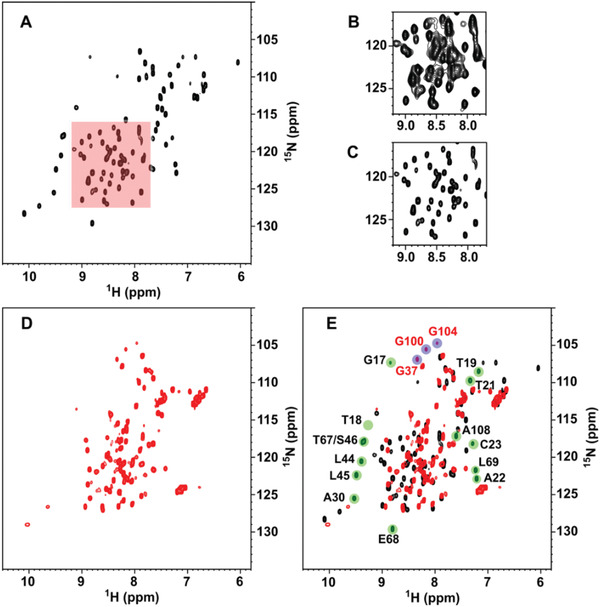
A–C) ^15^N‐^1^H HSQC NMR spectrum of SARS‐CoV‐2 Nsp9 (138 × 10^−6^ m in phosphate buffer, pH 7.03, 298 K). A moderately strong resolution enhancement weighing function (45°‐shifted squared sinebell) was applied prior to 2D Fourier transform. For the red‐highlighted region, the right panels show the difference between the signals without (B) and with (C) the same resolution enhancement as applied in (A). D) ^15^N‐^1^H HSQC NMR spectrum of SARS‐CoV‐2 triSer‐Nsp9 (131 × 10^−6^ m in aqueous acetate, pH 4.7, 298 K). Similar spectra are obtained also at pH 3.7 and 6.1. E) Overlay of the HSQC maps of triSer‐Nsp9 (red contours) and wild‐type Nsp9 (black contours) from SARS‐CoV‐2. Green highlighting marks the missing backbone amide cross‐peaks in the mutant spectrum, whereas blue highlighting indicates the missing connectivities in the wild‐type spectrum. The assignments of the missing signals in the spectrum of triSer‐Nsp9 are reported in black. The assignment of some of the additional signals present only in the spectrum of triSer‐Nsp9 is shown in red and is tentative for G100 and G104. All assignments were from Biological Magnetic Resonance Bank (BMRB 6501, 50 513, 50 622).

**Figure 3:**
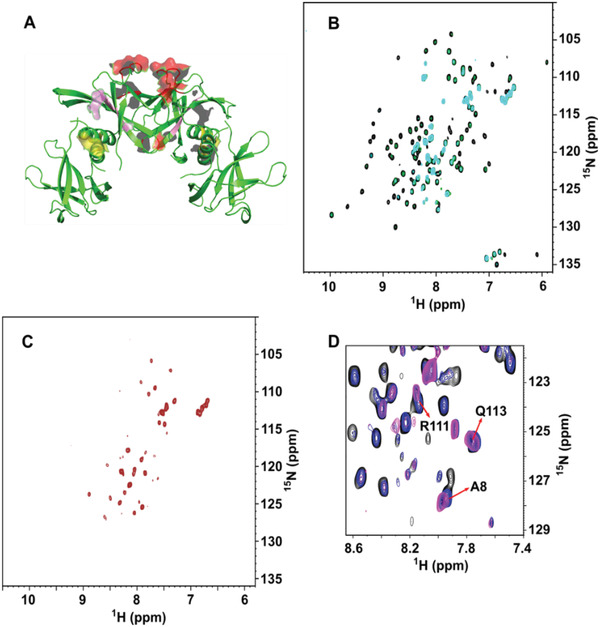
A) Crystal structure of SARS‐CoV‐2 Nsp9 tetramer (PDB ID: 7BWQ). The red regions locate fragments 67–69, 17–22, and residue 37, namely the interdimer contact surface that results highlighted by the different NMR pattern observed with triSer‐Nsp9 and Nsp9. The yellow regions show the positions of G100, G104, and A108 at the intradimer contact surface. Also, these residues exhibit a responsive pattern when comparing the spectra of the mutant and wild type. The pink regions (fragments 30–32 and 44–46) respond with a similar pattern as the red regions in the mutant spectrum, most likely revealing effects that occur more distantly with respect to the contact areas. B) Overlay of the ^15^N‐^1^H HSQC maps of SARS‐CoV‐2 Nsp9 recorded at 278 K, in the absence (black contours) and presence of 2NSP23, at protein:nanobody ratio 1:0.43 (green contours) and 1:0.63 (cyan contours). C) ^15^N‐^1^H HSQC spectrum of Nsp9 and 2NSP23 at protein:nanobody ratio 1:2. Similar patterns were obtained also with 2NSP90. D) Overlay of ^15^N‐^1^H HSQC regions of Nsp9 recorded at 276 K, in the absence (black contours) and presence of 2NSP90, at protein:nanobody ratio 1:0.43 (blue contours) and 1:0.74 (magenta contours). Analogous chemical shift changes were observed also with 2NSP23.

To begin characterizing the nature of the interaction of 2NSP90 and 2NSP23 with their antigen Nsp9, we performed in‐solution nuclear magnetic resonance (NMR) spectroscopy on wild‐type Nsp9 and triSer‐Nsp9. We first collected the ^15^N‐^1^H heteronuclear single quantum coherence (HSQC) NMR spectrum of SARS‐CoV‐2 Nsp9 (**Figure** **2**A). The quality of the spectrum does not match the expectation for a protein of ≈13.4 kDa, the mass of our ^13^C, ^15^N‐labeled Nsp9 construct. The cross‐peaks are broadened (Figure 2B,C) by the dimerization and possibly tetramerization that was anticipated from crystallographic evidence.^[^ ^5^, ^6^, ^7^ ^]^ This result is compatible with the recently reported NMR studies of SARS‐CoV‐2 Nsp9.^[^ ^11^, ^12^ ^] 2^H, ^15^N, ^13^C triply‐labeled and selectively labeled samples were in fact necessary to improve coherence transfer in 3D experiments for backbone assignment.^[^ ^11^ ^]^ Our 3D data confirmed poor coherence transfer for both wild‐type Nsp9 and mutated triSer‐Nsp9. Our HSQC maps also show that the backbone amide connectivities from the residues of the dimerization interface (segments 1–7 and 96–106) are largely missing due to the intermediate exchange, on the chemical shift scale, of the dimerization process.^[^ ^11^ ^]^ An even more severe loss of cross‐peaks affects the ^15^N‐^1^H HSQC spectrum of the triSer‐NSP9 mutant (Figure 2D,E). Apart from the obvious lack of C14, C23 and C73 cross‐peaks (replaced by serine ones), the signal loss of the mutant spectrum also concerns additional locations that significantly match the dimer–dimer interface of the tetramer.^[^ ^6^, ^7^ ^]^ Therefore, the triSer‐Nsp9 NMR spectrum reveals a further exchange implying the loss of the signals at the tetramerization interface because of an intermediate regime on the chemical shift scale. This is much like the dimerization exchange observed also in the wild‐type protein. In particular, cross‐peak loss is seen for the stretches 67–69 and 17–22 of interdimer contact surface, whereas the stretches 30–32 and 44–46, whose signals also disappear in the triSer‐Nsp9 spectrum, are located below that interface (**Figure** **3**A) and may report, therefore, the effect of a more distant conformational change related to tetramerization. Alternatively, this allosteric effect could reflect an additional response that maps to the monomer–monomer interface, as further inferred from the comparison of the HSQC spectra (Figure 2E). The onset of the three cross‐peak in the triSer‐Nsp9 spectrum with the typical chemical shifts of glycine amides suggests that two of these signals could be tentatively assigned to G100 and G104, whereas the third is from G37. The relative cross‐peak is, in fact, barely observed in wild‐type Nsp9 but becomes well visible in the mutated triSer‐Nsp9 probably because of dynamical changes induced by the proximity to the other dimer–dimer contact involving T35 and K36.^[^ ^6^ ^]^ The concurrent disappearance of A108 correlation in the triSer‐Nsp9 spectrum, together with the involvement of the N‐ and C‐terminal fragment, along with the G100XXXG104 motif in the intermonomer interface,^[^ ^6^, ^7^, ^13^, ^14^ ^]^ suggest some rearrangement of this interface upon tetramerization (Figure 3A). The higher extent of oligomerization in triSer‐Nsp9 was further confirmed by NMR diffusion ordered spectroscopy (DOSY) measurements of translational diffusion coefficient^[^ ^15^, ^16^ ^]^ (Figure S4, Supporting Information). To study how 2NSP23 and 2NSP90 interact with Nsp9, we collected HSQC spectra of ^15^N‐labeled wild‐type Nsp9 upon titration with an unlabeled nanobody.^[^ ^17^ ^]^ As already mentioned, the HSQC spectrum of Nsp9 (Figure 2A) shows the effect of the intermediate exchange between monomer and dimer that literally bleaches the amide cross‐peaks of the residues in contact at the dimerization interface.^[^ ^11^, ^12^ ^]^ To improve the signals in HSQC maps, we decreased the temperature to slow down the exchange. The overlay of the HSQC spectra of Nsp9 obtained at 298 and 278 K confirms that this was the case, for instance, with the increase of the intensities of T18, G17, G37, G61, and G63 cross‐peaks, which should improve the confidence of the analysis (Figure S5, Supporting Information). The titrations were therefore carried out at 278 K for 2NSP23 and 276 K for 2NSP90. Upon progressive addition of 2NSP23, an increasing number of amide cross‐peaks of the protein disappeared (Figure 3B), featuring the pattern expected for an intermediate exchange on the chemical shift scale. This intermediate exchange is typically observed when 200–300 × 10^−9^ m < *K*_D_ < 2–3 × 10^−6^ m, where *K*_D_ is the complex dissociation constant. By the end of the titration, at a protein/nanobody ratio of 1:2, only some 35% of the backbone amide cross‐peaks survive (Figure 3C). The same pattern was also observed with 2NSP90, with signal loss always preceded by progressing intensity attenuation. Table S3 (Supporting Information) lists the HSQC signals of Nsp9 that disappear as a function of the concentration of added nanobody.

**Figure 4:**
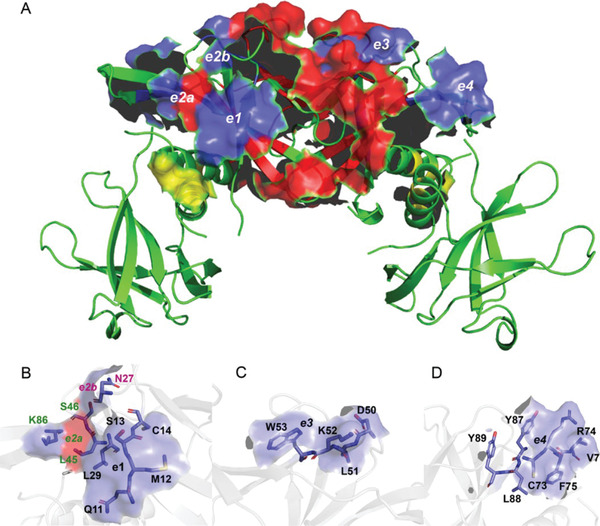
A) The SARS‐CoV‐2 Nsp9 tetramer with the interdimer and intermonomer contact surfaces highlighted in red and yellow, respectively. The blue surfaces indicate the location of the tetramer epitopes interacting with nanobodies 2NSP23 and 2NSP90. An analogous epitope pair is present on the opposite face of the tetramer. The first epitope is comprised of the surfaces e1, e2a, and e2b formed by segment [Q11‐M12‐S13‐C14] with residue L29, residue N27 and residue K86, respectively, B) and the additional contributions from L45 and S46 that are already part of the tetramer interface. The second epitope is comprised of the surfaces e3 and e4 formed by the segments C) [D50‐L51‐K52‐W53] and D) [C73‐R74‐F75‐V76 + Y87‐L88‐Y89], respectively.

In addition to the cross‐peak loss, also the rate of intensity attenuation before disappearance is a meaningful parameter suggesting that the two nanobodies are quite similar in the way they interact with Nsp9 (Table S3, Supporting Information). In particular, the residues with high attenuation rates and the order of peak loss replicate the regions involved directly and indirectly in the tetramer assembly and the dimerization interface rearrangement, namely 67–69, 17–22, 37, 30–32, 44–46, and 108 (**Figure** **4**A), with extensions including adjacent segments or single residues. However, some fragments of Nsp9 undergo fast attenuation and/or subsequent signal loss that appear unrelated to the tetramerization interface, namely at positions 11–14, 27, 29, 50–53, 73–76, 86–89. These fragments cluster on two accessible surface regions flanking the tetramerization interface and should represent the epitopes of the Nsp9 tetramer for both nanobodies (Figure 4). Each dimer of the Nsp9 tetramer contributes two epitopes on opposite faces, hence the epitopes on the same face of the tetramer (Figure 4A) are contributed by different dimers. The question arises on the number of nanobody monomers required to saturate the Nsp9 tetramer. A plausible stoichiometry for the Nsp9 tetramer could be four nanobody molecules. Evidence in favor of this stoichiometry comes from the fitting of the chemical shift variations observed for A8 and Q113 cross‐peaks along Nsp9 titration with 2NSP90 (Figure 3D), leading to statistically significant estimates of the number of nanobody‐binding sites—between 3 and 4—and the half occupation constant of ≈10 × 10^−6^ m (see Figure S6 in the Supporting Information). Besides the massive cross‐peak loss representing the progressive propagation of the intermediate exchange regime with titrant saturation, we could also detect progressive chemical shift changes associated with titration (Figure 3D). These involve mostly the N‐terminal and C‐terminal residue signals, namely A8, L9, R111, and Q113 and a couple of other locations (C73, V76). The pattern is compatible with the intermediate exchange regime observed for all the other residues of Nsp9 and may arise for intrinsically mobile molecular locations where the chemical shift is effectively averaged by the local dynamics, leading to a very small difference between the limiting chemical shift values and matching therefore local fast exchange regime.

## 3 Conclusions

In summary, we report the discovery of a novel cohort of nanobodies that specifically target the SARS‐Cov‐2 Nsp9 protein required for assembly of the RTC complex and, consequently, for viral replication. We demonstrate that two among these nanobodies, 2NSP23 and 2NSP90, specifically recognize Nsp9 in saliva samples collected from individuals infected with SARS‐Cov‐2 and may, therefore, be of potential diagnostic value for rapid identification and screening of COVID‐19. Further work is necessary, however, for an optimal diagnostic protocol that could be based on ELISA readout.

Structural characterization of 2NSP23 and 2NSP90 interactions by NMR analysis revealed that these nanobodies stabilize a tetrameric Nsp9 form. As this is not compatible with its monomeric configuration within the RTC complex,^[^ ^8^ ^]^ these nanobodies may contribute to the inhibition of viral replication by forcing the protein in a state that is not suitable for its RTC recruitment. Although RNA replication can also occur in vitro with a minimal complex formed only by Nsp7, Nsp8, and Nsp12,^[^ ^18^ ^]^ the requirement for Nsp9, first shown by a cryo‐EM structure of the RTC complex,^[^ ^8^ ^]^ appears furtherly established by increasing evidence on the NiRAN domain catalytic activity.^[^ ^19^ ^]^ Although further work is necessary, we speculate that 2NSP23 and 2NSP90 may serve as a possible Nsp9 inhibitor, negatively impacting on SARS‐CoV‐2 replication by perturbing the monomer–dimer–tetramer transition toward the induction of a stable tetramer.

## Conflict of Interest

GE and PP are part of a US provisional patent application filed by New York University in Abu Dhabi.

## Author Contributions

Y.H., M.P., and T.V. contributed equally to this work. G.E., P.P. conceptualized the research. Y.H., M.P., T.V., M.M.D., and G.H. conducted the experiments. F.F. did MD simulations and fitting statistics. F.P., K.C.G., and Y.I. provided key resources. G.E. and P.P. supervised the research. P.P. and G.E. wrote the manuscript. All authors read and approved the manuscript.

## Supporting information



Supporting information

## Data Availability

The data that support the findings of this study are available from the corresponding author upon reasonable request.
