# Supplementary material for: NMR‐Based Analysis of Nanobodies to SARS‐CoV‐2 Nsp9 Reveals a Possible Antiviral Strategy Against COVID‐19
Source: Adv Biol (Weinh). 2021 Oct 27;5(12):2101113. doi: 10.1002/adbi.202101113 (PMC8646926; doi:10.1002/adbi.202101113)
Supplement: Supplementary file 1 — Supporting information [file ADBI-5-2101113-s001.pdf]

Supporting Information

Supplemental Figures and Experimental Protocols

NMR-based analysis of nanobodies to SARS-CoV-2 Nsp9 reveals a possible antiviral strategy against COVID-19

Gennaro Esposito[1,2], Yamanappa Hunashal[1‡], Mathias Percipalle[1,3‡], Tomas Venit[4‡], Mame Massar Dieng[4], Federico Fogolari[5,2], Gholamreza Hassanzadeh[6], Fabio Piano[4,7], Kristin C. Gunsalus[8,9], Youssef Idaghdour[4,7], Piergiorgio Percipalle[4,10*]

[1]Chemistry Program, Science Division, New York University Abu Dhabi, Abu Dhabi 129188, United Arab Emirates; [2]Istituto Nazionale Biostrutture e Biosistemi, 00136 Roma, Italy; [3]Department of Chemistry and Magnetic Resonance Center, University of Florence, 50019 Florence, Italy; [4]Biology Program, Science Division, New York University Abu Dhabi, Abu Dhabi 129188, United Arab Emirates; [5]Dipartimento di Scienze Matematiche, Informatiche, e Fisiche, Udine Univer-sity, 33100 Udine, Italy; [6]VIB Nanobody Core, Vrije Universiteit Brussel, 1050 Brussels, Belgium; [7]Public Health Research Center, New York University Abu Dhabi, Abu Dhabi 129188, United Arab Emirates; [8]Center for Genomics and Systems Biology, New York University Abu Dhabi (NYUAD), P.O. Box 129188, Abu Dhabi, United Arab Emirates; [9]Department of Biology, Center for Genomics and Systems Biology New York University, New York, NY, 10003, USA; [10]Department of Mo-lecular Bioscience, The Wenner Gren Institute, Stockholm University, Stockholm, Sweden

‡ Equal contributions

[*]Correspondence: Piergiorgio Percipalle, address as above

Tel.: +97126284389

Email: pp69@nyu.edu

‡ These authors equally contributed to the work

* Corresponding author

Contents

I.	Experimental procedures	4-14
II.	References	15-17
III.	Supplementary Figures and Tables	18-30

I. Experimental procedures

triSer-Nsp9 protein preparation. Recombinant wild-type (wt) Nsp9 and triSer-Nsp9 proteins carrying the three amino acid substitutions C14S, C23S, C73S were provided by ASLA Biotech (Lot No. 2007ZJ03NSP01). Unlabeled and uniformly ^{15}N,^{13}C-labelled SARS-CoV-2 C14S,C23S,C73S NSP9 (triSer-NSP9) was expressed with an additional methionine at the N-terminus (M0). A construct was used for expression where the his-tag is located after the stop codon and therefore is not expressed. The scheme for this genetic construct was: pET22b(NdeI)-NSP9(113 aa + M0)-pET22b(XhoI) where CATATG is for NdeI; CTCGAG for XhoI; TAA is a stop codon, corresponding to DNA sequence:

CATATGAACAACGAACTGAGTCCGGTGGCACTGCGTCAGATGAGTAGTGCCGCCGGCACC
ACCCAGACCGCCAGTACAGATGATAATGCCCTGGCCTATTATAATACCACCAAAGGTGGTC
GCTTTGTTCTGGCACTGCTGAGCGATCTGCAGGATCTGAAATGGGCACGTTTTCCGAAAAG
CGATGGTACCGGCACCATCTATACCGAACTGGAACCGCCGAGTCGTTTTGTGACCGATAC
CCCGAAAGGTCCGAAAGTGAAATATCTGTATTTTATTAAGGGTCTGAACAACCTGAATCGT
GGTATGGTGCTGGGTAGTCTGGCAGCAACCGTTCGCCTGCAGTAACTCGAGCACCACCAC
CACCACCACTGA.

After IPTG induction of the Escherichia coli BL21 (DE3) transformed strain, the cells were grown at 22°C for 18 hours in M9 medium containing 13C-labeled glucose and 15N-labeled ammonium chloride. The cell pellet was collected by centrifugation and resuspended in lysis buffer (1/40 volume: 1xPBS, protease inhibitor cocktail), sonicated and then centrifuged at 32000xg for 30 min. The cell pellet was dissolved in 6M GuCl,

50mM NaPi, pH 7.3 solution and incubated for 40 min. The cell lysate was again centrifuged at 32000xg. The recombinant protein was further purified by cation exchange chromatography using a HiTrap SP HP column (GE Healthcare, 20 ml). The protein rich fractions were pooled, concentrated and applied to a Superdex 200 size-exclusion column (GE Healthcare, 120 ml). The protein was refolded by drop dilution into refolding buffer (100mM Tris, 5mM EDTA, 0.75M arginine, pH 8) on ice. The protein was further dialyzed twice against PBS (1:40 volume), and then again concentrated and dialyzed by Amicon Ultra 15 (Merck Millipore) against 20 mM ammonium acetate. The final protein preparation was lyophilized.

The uniformly ^{15}N,^{13}C-labelled wild-type SARS-CoV-2 NSP9 was obtained according to the protocol kindly provided by the NMR COVID-19 Consortium, [1] leading to a final product with an additional GlyAlaMetGly tetrapeptide at the N-terminus.

For immunization, about 3 mg of lyophilized triSer-Nsp9 protein were dissolved in 20 mM ammonium acetate (pH 6.7). As the lyophilized protein was not fully dissolved in this buffer, the supernatant obtained by centrifugation, was recovered and stored for further use in immunization, panning and ELISA screening. The protein pellet (insoluble protein fraction) was dissolved in a small amount of dimethyl sulfoxide (DMSO), and the protein in DMSO was immediately diluted in PBS. Both the supernatant obtained by dissolving the protein in ammonium acetate, followed by centrifugation, and the protein dissolved in DMSO & diluted in PBS were used separately in the immunization, panning and ELISA screening experiments described below. In these experiments, we refer to the protein in

ammonium acetate as NSP, and the protein dissolved in DMSO and diluted in PBS as INS.

Immunization. A llama was subcutaneously injected on days 0, 7, 14, 21, 28 and 35, each time with about 100 µg of recombinant triSer-NSP9 dissolved in ammonium acetate (here referred to as NSP) & 100 µg of recombinant triSer-NSP9 protein dissolved in DMSO/PBS (here referred to as INS). Each injection was performed at 2 sites at the end of the neck (cranially or caudally to the scapula bone) and at 2 sites at the two back limbs. The protein dissolved in ammonium acetate (NSP) was injected on the left side of the animal and the protein dissolved in DMSO/PBS (INS) was injected on the right side of the animal. The adjuvant used was Gerbu adjuvant P. On day 40 (5 days after last immunization), about 100 ml anticoagulated blood was collected from the llama for peripheral blood lymphocytes (PBLs) preparation and library generation.

Construction of a VHH (Nanobody) library. A VHH library was constructed from PBLs to screen for the presence of antigen-specific nanobodies. To this end, total RNA was prepared from PBLs and about 50 µg of total RNA was used as template for first strand cDNA synthesis with oligodT primers. Using this cDNA, the VHH encoding sequences were amplified by PCR, digested with PstI and NotI, and cloned into the PstI and NotI sites of the phagemid vector pMECS. In pMECS vector, the nanobody sequence is followed by a linker, HA tag and His_6 tag (Nanobody-AAAYPYDVPDYGSHHHHHH). Electro-competent E. coli TG1 cells were transformed with the recombinant pMECS vector resulting in a VHH (nanobody) library of about 2 x 10^9 independent transformants. This library is named “Core 145/146 library”. About 87% of the transformants from this

library harbored the vector with the right insert size (size of VHH-encoding sequences), as demonstrated by PCR analysis of 95 randomly picked colonies.

Nanobodies isolation. The Core 145/146 library was panned separately on NSP & INS protein samples immobilized on solid-phase (100 µg/ml in 100 mM NaHCO3 pH 8.2). Three rounds of panning were performed on each protein sample. The enrichment for antigen-specific phages was assessed after each round of panning by comparing the number of phagemid particles eluted from antigen-coated wells with the number of phagemid particles eluted from negative control (uncoated blocked) wells.

For panning on protein sample dissolved in ammonium acetate (NSP), the phage population was enriched for antigen-specific phages about 20-fold, 100-fold and 700-fold after the 1st, 2nd and 3rd round, respectively. In total, 190 colonies from 2nd and 3rd rounds (95 colonies from each round) were randomly selected and analyzed by ELISA for the presence of antigen-specific Nanobodies in their periplasmic extracts (ELISA using crude periplasmic extracts including soluble Nanobodies). The ELISA tests were performed on both protein dissolved in ammonium acetate (NSP) and the protein dissolved in DMSO/PBS (INS). Uncoated blocked wells served as negative control (blank) for ELISA. Out of these 190 colonies, 165 colonies scored positive for NSP and/or INS. Based on sequence data of the 165 positive colonies, 71 different Nanobodies were identified, belonging to 18 different CDR3 groups (B-cell lineages). Out of these 71 Nanobodies, 65 Nanobodies are positive for both NSP and INS, while 6 Nanobodies bind only to NSP (See Excel file). The 71 different nanobodies specific for Nsp9 which resulted from panning on protein dissolved in ammonium acetate bear the code “NSP” in their names.

For panning on protein sample dissolved in DMSO/PBS (INS), the phage population was enriched for antigen-specific phages about 30-fold and 80-fold after the 2nd and 3rd round, respectively. No clear enrichment was observed after the 1st panning round. Here also, 190 colonies from 2nd and 3rd rounds (95 colonies from each round) were randomly selected and analyzed by ELISA for the presence of antigen-specific Nanobodies in their periplasmic extracts (ELISA using crude periplasmic extracts including soluble Nanobodies). The ELISA tests were performed on both the protein dissolved in ammonium acetate (NSP) and the protein dissolved in DMSO/PBS (INS). Uncoated blocked wells served as negative control (blank) for ELISA. Out of these 190 colonies, 156 colonies scored positive for INS and/or NSP. Based on sequence data of the 156 positive colonies, 65 different nanobodies were identified, belonging to 22 different CDR3 groups (B-cell lineages). Out of these 65 nanobodies, 59 nanobodies are positive for both INS and NSP, while 2 nanobodies are only positive for INS and 4 nanobodies bind only to NSP (See Excel file). The 65 different nanobodies specific for SARS-CoV-2 Nsp9 which resulted from panning on protein dissolved in DMSO/PBS bear the code “INS” in their names.

In summary, the above-described experiments resulted in 136 different nanobodies, belonging to 40 different CDR3 groups (B-cell lineages). Out of these 136 nanobodies, 124 specifically recognize both protein dissolved in ammonium acetate and protein dissolved in DMSO/PBS, while 12 nanobodies recognize either of these protein samples. The most likely explanation for differential binding of the later 12 nanobodies to 2 different protein samples is the experimental variabilities since the ELISA data here are all from single experiments. This is strongly supported by the fact that within the same CDR3

group (the same target epitope), there are both nanobodies binding to two different protein samples and also nanobodies which bind only to one protein sample. nanobodies belonging to the same CDR3 group (same B-cell lineage) are very similar and their amino acid sequences suggest that they are from clonally-related B-cells resulting from somatic hypermutation or from the same B-cell but diversified due to RT and/or PCR error during library construction. Nanobodies belonging to the same CDR3 group recognize the same epitope but their other characteristics (e.g. affinity, potency, stability, expression yield, etc.) can be different.

Expression and purification of nanobodies. Each nanobody was expressed in about 2L of TB medium (6 x 330 ml). Small scale overnight cultures were started in 20 mL of LB medium supplemented with ampicillin (100 µg/ml) and glucose (1%). The overnight cultures were used to inoculate the shaker flasks, each containing 330 mL of TB medium supplemented with ampicillin (100 µg/ml) and glucose (0.1%), and grown at 220 rpm at 30°C. After the cultures reached an OD_{600}nm of about 0.8, expression was induced by addition of IPTG to final concentration of 1 mM. The induced cultures were then incubated at 20°C at 220 rpm. The following day, the cultures were spun down. The supernatants were discarded and the cell pellets were subject to osmotic shock by resuspending each pellet from 330 ml culture in 4 mL TES buffer & incubating for 2 h at 4°C with gentle shaking, followed by addition of 8 mL of water and further 4 h incubation at 4°C. The periplasmic extracts (PEs) were collected by centrifugation and stored at 4°C. The cell pellets were used for a second osmotic shock cycle. When all extracts were collected, 500 µL of His-select matrix was added to each individual periplasmic extract (PE) and incubated for 1 h at 4°C. Each PE was then applied to an empty PD-10 column. The flow

through was applied to the column for a second time. Subsequently, the matrix was washed with a solution of 20 mM imidazole in PBS. Finally, the bound Nanobodies were eluted using a solution of 500 mM imidazole in PBS, in 5 fractions of 1 mL. These fractions were monitored by Nanodrop at 280 nm to quantify the amount of nanobody collected. The fractions containing substantial amounts of protein were pooled and loaded onto a Superdex 75 16/60 or a Superdex 75 10/300 GL Increase size exclusion chromatography (SEC) columns.

Saliva samples collection and storage. Saliva samples from individuals with known SARS-CoV-2 status (positive and negative) were used for this study. Approximately 1 ml of saliva samples were collected in sterile 5 ml falcon tubes (Fisher scientific) without transport medium under NYUAD IRB-approved protocol HRPP-2020-48 (PI Idaghdour), stored directly at 4°C until shipment to a BSL2 laboratory for processing following guidelines from the US Center for Disease Control and Prevention (CDC). The samples were stored at -80°C until processing.

Saliva RNA isolation and SARS-CoV-2 Detection by RT-qPCR. For each sample, 300µl of saliva were used to extract RNA in a BSL2 laboratory using an automated system, Chemagic 360, and Viral DNA/RNA 300 Kit H96, both from Perkin Elmer. Extracted RNA was eluted in 80 µL of elution buffer and used right away for SARS-CoV-2 detection or stored at -80°C until use as per manufacturer's instruction protocol. The full protocol for SARS-CoV-2 detection using RT-qPCR and the Fluidigm BioMark HD system is described in Xie *et al*, 2020. [2] CDC recommended assays (primers and probes) for SARS-CoV-2 detection, and human RNase P (RP) control for RNA extraction and RT-

qPCR reactions were used. The assays were synthesized by Integrated DNA Technology (IDT) and the sequences are as follow: 2019-nCoV_N2 (Forward: TTA CAA ACA TTG GCC GCA AA; Reverse: GCG CGA CAT TCC GAA GAA; Probe: ACA ATT TGC CCC CAG CGC TTC AG); human RNase P (RP) (Forward: AGA TTT GGA CCT GCG AGC G; Reverse: GAG CGG CTG TCT CCA CAA GT; Probe: TTC TGA CCT GAA GGC TCT GCG CG). For each sample, five microliters of extracted RNA were reverse transcribed (RT) to cDNA using 1.25 µL RT Master Mix following the manufacturer protocol (Fluidigm). The converted cDNA was pre-amplified using a pool of the three assays (N1, N2, and RP) diluted into low TE buffer (Thermo Scientific) to a final concentration of 100.5 and 25.5 nM for the primers and probes, respectively, and then mixed with 2.5 µL of Preamplification Master Mix, and 0.635 µL PCR grade water (Fluidigm). After the pre-amplification step, the PCR reactions were further diluted 1:5 in Low TE buffer (Thermo Scientific) resulting in a final volume of 62.5 µL and ready to use for qPCR. The qPCR mix was prepared using 1.8 µL of diluted cDNA and 2 µL of 2X TaqMan Fast Advanced Master Mix (Thermo Scientific) and 0.2 µL 20X GE Sample Loading Reagent (Fluidigm). Next, 3 µL qPCR mix from each sample was loaded into the sample inlet in the 192.24 integrated fluid circuits (IFC, Fluidigm). For each assay, 3 µL of primer/probe mix (13.5X) was mixed with 1 µL of 4X Assay Loading Reagent (Fluidigm), and 3 µL of each assay mixed was loaded in the assay inlet in the 192.24 or Flex Six IFC chip (Fluidigm). The chip was then placed in an integrated fluidic circuit (IFC) controller RX machine to pre-load the samples and the assays, and then, loaded onto the BioMark HD instrument (Fluidigm) for RT-qPCR using 35 cycles. The raw amplification data were acquired using the Fluidigm data collection software and analyzed using the Fluidigm Real-Time PCR

Analysis software 3.0.2. The expression of viral N2 protein was normalized to human RNase P expression and compared between samples. Each RT-qPCR reaction was repeated at least 4 times.

Western blot analysis. Purified wtNsp9 was diluted in 1xPBS buffer to prepare 1mg/ml stock solution with protease inhibitors. The stock wtNsp9 solution was further diluted with 1xPBS containing 1mg/ml bovine serum albumin (BSA) to the final concentration of 15 µg of total protein per loading with decreasing amount of wtNsp9 per sample. Following SDS PAGE, protein samples were visualized by PageBlue Protein Staining solution (Thermo Fisher) according to the manufacturer's instructions. For preparation of saliva samples, 20µl of saliva was mixed with 2x RIPA buffer (50 mM Tris-HCl pH 7.5, 150 mM NaCl, 1 mM EDTA, 1 % NP-40, 0.5 % sodium deoxycholate, 0.1 % SDS) in 1:1 ratio and total protein concentration measured by the Pierce BCA Assay Kit (Thermo Fisher Scientific). Subsequently 150 µl of the saliva sample was mixed with 50 µl of 4x Laemmli buffer with protease inhibitors and based on the protein concentration measurement by BCA assay, approximately 15µg of total protein per sample was loaded to the 15% SDS-PAGE gel. The extracts were separated under reducing conditions and transferred to polyvinylidene difluoride (PVDF) membrane and blocked with 3% BSA in 1xTBST buffer (20 mM Tris, 150 mM NaCl, 0,1% Tween 20) for 1 hour. After blocking, membranes were incubated with nanobodies 2NSP23 and 2NSP90 (dilution 1000x in 1xTBST buffer) overnight at 4°C and subsequently washed 4 times for 15 minutes in 1xTBST buffer. Immunoblots were then stained with HRP-conjugated secondary antibodies (dilution 2000x in 1xTBST buffer) recognizing either 6xHis (ab237339, Abcam) or VHH epitopes

(128-035-230, Jackson ImmunoResearch). Detection was by chemiluminescence using the ECL Western Blot Substrate (Bio-Rad) and imaged by a ChemiDoc MP Imaging system (Bio-Rad).

NMR spectroscopy. TCEP (tris(2-carboxyethyl)phosphine), sodium phosphate, hen egg white lysozyme (HEWL), D_2O and DMSO-d6 were all from Sigma Aldrich (St. Louis, MO, USA). Perdeuterated glycine was from Cambridge Isotope Laboratories (Tewksbury, MA, USA). The uniformly ^{15}N,^{13}C-labelled wild-type SARS-CoV-2 Nsp9 and triSer-Nsp9 were obtained as above described in Experimental Protocols. The wild-type protein samples were typically prepared in H_2O/D_2O 95/5 with phosphate buffer 10-30 mM, 60-150 mM NaCl, 0.4-1.2 mM TCEP, 0.004-0.01% NaN_3, pH 7.0-7.1. For the triSer-Nsp9 samples, additional buffers and pH conditions were explored besides the phosphate (25 mM with 75 mM NaCl, pH 7.0). In particular triSer-Nsp9 was first prepared and lyophilized from 20 mM ammonium acetate solution. Upon redissolving in H_2O/D_2O, pH was 6.1 (residual salt was observed) and the quality of the spectra was poor. Addition of saline phosphate buffer resulted in pronounced precipitation. This was also the case with the protein lyophilized from pure water that exhibited better solubility when dissolved without further additions (pH 4.7), or after dialysis against water (pH 3.7), or with 5% addition (in volume) of DMSO-d6, or in 100 mM perdeuterated glycine, pH 6.1. Under all these conditions, no major differences were detected with respect to phosphate, except for the solubility. In general, for the protein samples, concentrations ranged from 18 to 431 μM, as determined by UV absorption at 280 nm. No relevant difference was observed at different concentrations. For titrations with nanobodies (2NSP23 and 2NSP90) two mother solutions of 18 μM ^{15}N,^{13}C-labeled NSP9 without and with 36 μM unlabeled nanobody were progressively

mixed. The buffer was 10 mM phosphate, 150 mM NaCl, 0.4 mM TCEP, 0.004% NaN_3 with pH 7.2. No precipitation was observed during the whole titrations. The NMR experiments of the isolated proteins were mostly acquired at 298 K. With triSer-NSP9 some experiments were also recorded at higher temperatures (305, 310 and 315 K) in the absence and presence of 5% DMSO-d6 to check for linewidth improvements. For titration of the wild-type sequence with nanobodies, the temperature was lowered to 278 and 276 K before starting the experiments which were in fact conducted at those two temperatures with 2NSP23 and 2NSP90, respectively. The NMR data were collected at 14.0 T (^{1}H at 600.19 MHz, ^{15}N at 60.82 MHz) on a Bruker Avance III NMR system equipped with triple resonance cryoprobe. Two-dimensional ^{15}N-^{1}H HSQC experiments [3] were carried out using sensitivity-improved Echo/Antiecho-TPPI pure phase detection in F1, gradient coherence selection and flip-back pulse for solvent suppression [4-6]. Data were collected over 40-50 ppm and 12-16 ppm windows in F1 and F2, respectively, using 128 points in t_1 and 2,048 points in t_2, with 32, 64 or 256 scans/ t_1 increment and 16 or 32 dummy scans to achieve steady state. Prior to 2D Fourier transform, squared sine-bell apodization, shifted at least by $\pi/4$ in either dimensions, and linear prediction to double the data size in t_1 were applied, with zero filling to obtain 2Kx1K matrices of reals. ^{13}C was decoupled by band-selective adiabatic π pulses applied at the center of t_1 evolution. [7] DOSY [8] spectra for determination of diffusion coefficients were acquired by 2D ^{1}H DSTEBPP (Double STimulated Echo BiPolar Pulse) experiments with compensation for convective motions. [9] The z-axis gradient strength was varied linearly from 20 to 90% of its maximum value (~ 60 G/cm) and matrices of 2,048 by 40 points were collected by accumulating 32-64 scans per gradient increment. Water suppression

was achieved by appending to the DSTEBPP sequence a pair of WATERGATE [10] elements in the excitation-sculpting mode [11], as for 1D experiments. Following Fourier transform of the 1D traces, DOSY data fitting to extract the diffusion coefficients was carried out using Dynamics Center (Bruker). The related hydrodynamics calculations were done according to Hansen [12].

MD simulations. The dimer of SARS-COV-2 Nsp9 RNA-Replicase was taken from the structure deposited in the Protein Data Bank (pdb id: 6w4b). The mutation of all cysteines into serines was performed using the program DeepView4.10. [13] The structures were soaked in a box of TIP3P water [14] and 0.150 M NaCl up to at least 14 Å from any solute atom using the program VMD. [15] All molecular simulations were performed using the program NAMD2. [16] First each system was energy minimized by 5,000 steepest descent steps. The dynamics was started at 0 K and the temperature was increased to the target value in 10 ps and was equilibrated for 1 ns rescaling every 0.1 ps. During this phase and in all simulations, pressure was kept constant at 1.0 atmosphere by the Langevin piston method with period 200 ps and decay time 100 ps, at the target temperature. [17,18] In all simulations, except for the heating phase described above, the temperature was controlled through Langevin dynamics with damping constant 2.5 ps^{-1}. Interactions were gradually switched off at 12 Å starting at 10 Å. The time step was 1 fs for bonded interactions and 2 fs for nonbonded interactions. Hydrogen bond lengths were restrained by the algorithm Settle. [19] Exactly the same protocol was applied to the wildtype and mutant protein. Simulations were performed for 200 ns.

II. References

[1] E. F. Dudás, R. Puglisi, S.M. Korn, C. Alfano, M.L. Bellone, F. Dal Piaz, G. Kelly, E. Monaca, A. Schlundt, H. Schwalbe, A. Pastore, *Biomol. NMR Assign.* **2021**, doi:10.1007/s12104-021-10011-0.

[2] X. Xie, T. Gjorgjieva, Z. Attieh, M. M. Dieng, M. Arnoux, M. Khair, Y. Moussa, F. Al Jallaf, N. Rahiman, C. A. Jackson, L. El Messery, K. Pamplona, Z. Victoria, M. Zafar, R. Ali, F. Piano, K. C. Gunsalus, Y. Idaghdour, *Processes* **2020**, *8*, 1425.

[3] G. Bodenhausen, D. J. Ruben, *Chem. Phys. Lett.* **1980**, *69*, 185.

[4] A. G. Palmer, J. Cavanagh, P. E. Wright, M. Rance, *J. Magn. Reson.* **1991**, *93*, 151.

[5] J. Schleucher, M. Schwendinger, M. Sattler, P. Schmidt, O. Schedletzky, S. J. Glaser, O. W. Sørensen, C. Griesinger, *J. Biomol. NMR* **1994**, *4*, 301.

[6] S. Grzesiek, A. Bax, *J. Am. Chem. Soc.* **1993**, *115*, 12593.

[7] D. Rosenfeld, S. H. Panfil, Y. Zur, *J. Magn. Reson.* **1997**, *126*, 221.

[8] K. F. Morris, C. S. Johnson, *J. Am. Chem. Soc.* **1992**, *114*, 3139.

[9] A. Jerschow, N. Müller, *J. Magn. Reson.* **1998**, *132*, 13.

[10] M. Piotto, V. Saudek, V. Sklenár, *J. Biomol. NMR* **1992**, 2, 661.

[11] T. L. Hwang, A. J. Shaka, *J. Magn. Reson. A* **1995,** 112, 275.

[12] S. Hansen, *J. Chem. Phys.* **2004**, *121*, 9111.

[13] T. Schwede, J. Kopp, N. Guex, M. C. Peitsch, *Nucl. Ac. Res.* **2003**, *31*, 3381.

[14] W.L. Jorgensen, J. Chandrasekhar, J.D. Madura, R.W. Impey, M.L. Klein, *J. Chem. Phys.* **1983**, *79*, 926.

[15] W. Humphrey, A. Dalke, K. Schulten, *J. Mol. Graph.* **1996**, *14*, 33.

[16] L. Kale, R. Skeel, M. Bhandarkar, R. Brunner, A. Gursoy, N. Krawetz, J. Phillips, A. Shinozaki, K. Varadarajan, K. Schulten, *J. Comp. Phys.* **1999**, *151*, 283.

[17] G. Martyna, D. Tobias, M. Klein, *J. Chem. Phys.* **1994**, *101*, 4177.

[18] S. Feller, Y. Zhang, R. Pastor, B. Brooks, *J. Chem. Phys.* **1995**, *103*, 4613.

[19] S. Miyamoto, P. A. Kollman, *J. Comp. Chem.* **1992**, *13*, 952.

[20] C. Zhang, Y. Chen, L. Li, Y. Yang, J. He, C. Chen, D. Su, *Mol. Biomed.* **2020**, *1*, 5, doi:10.1186/s43556-020-00005-0.

III. Supplementary figures and tables

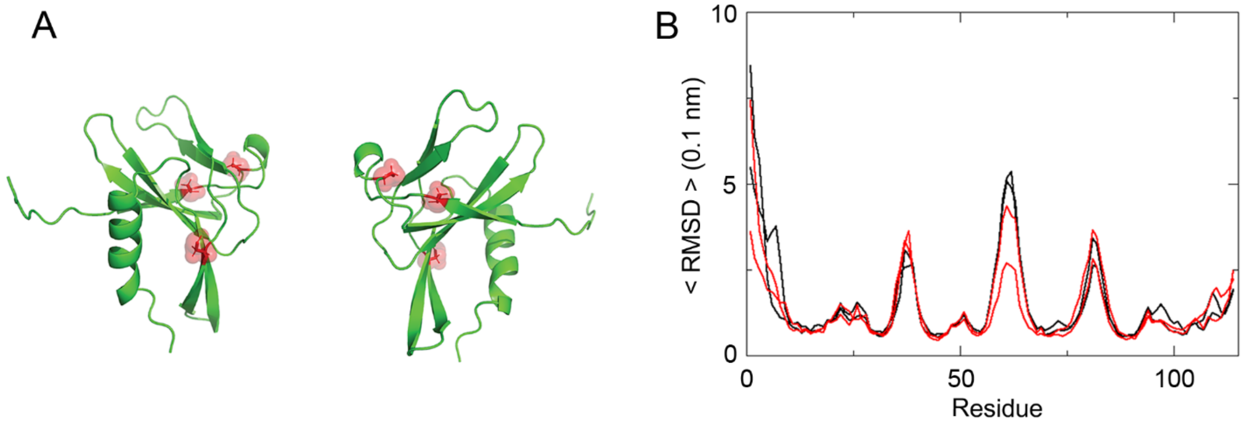

Figure S1. (A) Model of triSer-Nsp9 with red highlights at the positions of the three serines replacing the cysteines in the wild-type sequence. (B) Average pairwise RMSD values upon monomer-monomer superposition along the sequences of the homodimeric subunits of WT SARS-CoV-2 Nsp9 (red) and the triSer-Nsp9 mutant (black), calculated from snapshots selected every 1 ns from a 200 ns molecular dynamics (MD) trajectory. The two subunits of each homodimer are indicated by separate traces. Deviations were observed for one or both subunits of the wild-type homodimer at approximately residues 55-65, as well as at the N- and C- termini, corresponding to a slightly larger conformational spread of wild-type with respect to the corresponding dispersion of the mutant. Overall, however, the observation of a similar MD pattern confirmed that no major difference should occur between the parent and the mutant subunits of the homodimers. Therefore, the triSer-Nsp9 was deemed suitable for raising the HCAbs response.

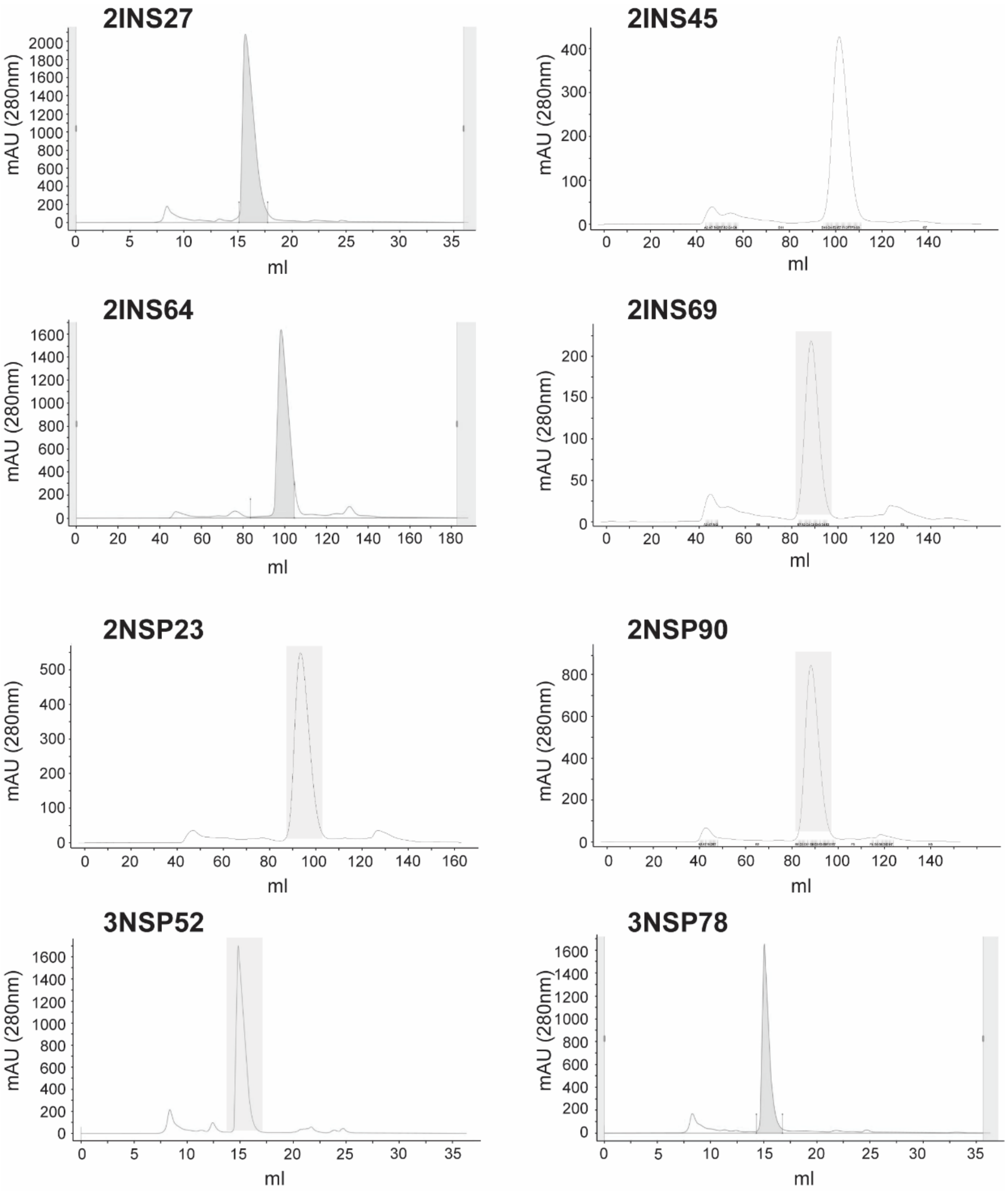

Figure S2. Representative size exclusion chromatogram profiles of selected nanobodies. The highlighted peaks represent the elution of each nanobody and the respective fractions collected.

A

```
                     CDR1            CDR2
3NSP52      QVQLQESGGGLVQPEGSLRLSCAASGLTFDSYAIGWFRQAPGKEREFVAAS-IWSGDSGHYTGSVKGRFTISRD 73
2NSP23      QVQLQESGGGLVQPGGSLRLSCAASGLAFSMYTMGWFRQAPGKEREFVAMI-ISSGDSTDYADSVKGRFTISRD 73
2INS69      QVQLQESGGGLVRAGDSLRLSCAVSGRTFSSVAMGWFRQAPGKEREFVAFISGSTGSVTYYADSVKGRFAISRD 74
2NSP90      QVQLQESGGGLVQTGDSLRLSCAVSGRTFSTYSVGWFRQAPGKEREFVAL--RWSGGTTYYADSVVGRFTVSRD 72
3NSP78      QVQLQESGGGLVQAGGSLRLSCAASGRAFSSYVMGWFRQAPGKEREFVAAI-HWTGAATVYVDSVKGRFAISRH 73
2INS27      QVQLQESGGGLVQAGGSLRLSCATSGSIFSSAVMGWYRQAPGNERELVALI--GSSDTTDYSNSVKGRFTISRD 72
2INS45      QVQLQESGGGLVQAGGSLRLSCAASGFTLSSNDMGWYRQAPGKQRELVATI--TSGLSTNYADSVKGRFTISRD 72
2INS64      QVQLQESGGGLVQVGGSLRLSCAASGSILSINAMGWYRQAPGKQRELVAAI--TSGGSTNYADSVKGRFTISRD 72
            ************:  .*******.**  :.   :**:*****::**:**      :.    * .** ***::**.

                                         CDR3
3NSP52      NAKNTVDLQMNSLKPEDTAVYYCAASASFLHSANYHMRAKWGYWGQGTQVTVSSAAAYPYDVPDYGSHHHHHH146
2NSP23      NGKNTVYLQMDSLKPEDTAVYYCAAPKFRYYF--STSPGDFDSWGQGTQVTVSSAAAYPYDVPDYGSHHHHHH144
2INS69      NAKNTVYLQMNSLKPEDTAVYDCAAKF-TGTF--NY-QGLYDYWGQGTQVTVSSAAAYPYDVPDYGSHHHHHH143
2NSP90      NAKNTVYLEMNSLKPEDTAVYYCAADRGSGSY--SP-TYRWDYWGQGTQVTVSSAAAYPYDVPDYGSHHHHHH142
3NSP78      NAKNTVYLEMNSLKPEDTAVYYCAADP-SGSY--WP-PKRYDYWGQGTQVTVSSAAAYPYDVPDYGSHHHHHH142
2INS27      NAKNTAYLRMNSLKPEDTAVYYCTAVKYG--------LGGFVYWGQGTQVTVSSAAAYPYDVPDYGSHHHHHH137
2INS45      NAKNTVFLQMNSLKIEDTAVYYCEVERGW--------GPPRDYWGHGTQVTVSSAAAYPYDVPDYGSHHHHHH137
2INS64      NAKNMLYLQMNSLKPEDTAVYYCHVVTGW--------GP--LDWGQGTQVTVSSAAAYPYDVPDYGSHHHHHH135
            *.**   *.*:*** ****** *  .                **:***************************
```

Green - glycine, thiol, hydroxyl, amine; **Red** - small, hydrophobic; **Blue** - acidic; **Magenta** - Basic
* - conserved position   : - strong positive identity   . - weak positive identity

B

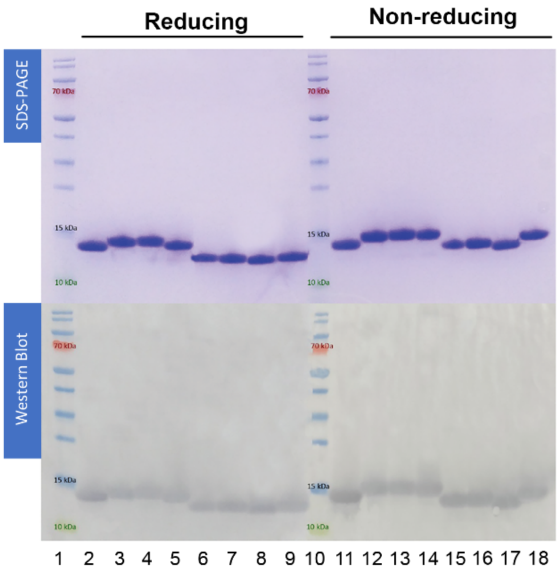

1 & 10 - PageRuler™ prestained protein ladder;
2 & 11 - 2NSP23; **3 & 12** - 2NSP90; **4 & 13** - 3NSP52; **5 &14** - 3NSP78; **6 & 15** - 2INS27;
7 & 16 - 2INS45; **8 & 17**; 2INS64; **9 & 18**; 2INS69

Figure S3. (A) Annotated amino acid sequences of eight selected nanobodies based on their DNA sequences. Alignment and annotations were done using the ProtParam tool (https://web.expasy.org/protparam/). (B) SDS-PAGE (top panels) and Western blot (bottom panels) analysis of the 8 purified nanobodies. Western blotting was performed with anti-His tag antibodies. For quality control, 5µg of each purified nanobody were loaded onto two 12.5% SDS-PAGE gels for Coomassie Blue staining or for Western blotting with mouse anti-His tag antibodies detected with a goat anti-mouse-HRP antibody. All purified Nanobodies revealed a single band profile on both gel and Western blot in both reducing and non-reducing conditions.

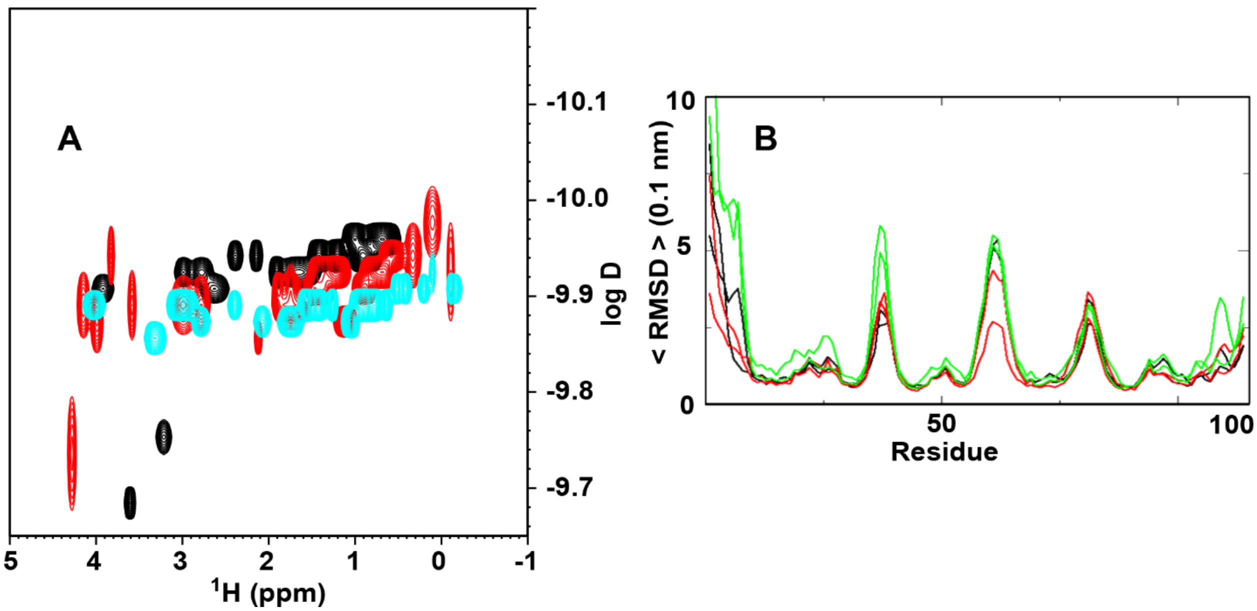

Figure S4. (A) Aliphatic region overlay from the DOSY contour maps of SARS-CoV-2 triSer-Nsp9 (black), wild-type Nsp9 (red) and hen egg white lysozyme (HEWL, cyan). The *D* values are $1.07 \pm 0.01 \times 10^{-10}$ m^2/s and $1.13 \pm 0.02 \times 10^{-10}$ m^2/s for the mutant and wild-type Nsp9, respectively. For HEWL, considered as a marker, a value of $1.24 \pm 0.02 \times 10^{-10}$ m^2/s is obtained. Protein concentrations in H_2O/D_2O 95/5 were ~ 130 μM for Nsp9 variants and 120 μM for HEWL. The overlay shows that the translational diffusion is slower for triSer-Nsp9 (black trace) compared to wild-type Nsp9 (red trace). Considering the crystallographic Nsp9 dimensions [20], the prolate ellipsoids of revolution representing monomers and dimers (with axial ratios 1.304 and 1.805, respectively) correspond to spheres with radius of 1.71 nm and 2.28 nm [12] with an expected D value of 1.06×10^{-10} m^2/s and 1.41×10^{-10} m^2/s, respectively. Therefore, the experimentally determined D values and the reported NMR evidence consistently indicate that the extent of oligomerization is more pronounced for triSer-Nsp9, with an essentially complete dimerization for the visible signals. Instead, the observable NMR signals of wild-type Nsp9 also bear some contribution from monomers, which indicates a difference in the extent of dimerization between triSer-Nsp9 and the natural sequence with three cysteines. (B) Average pairwise RMSD values upon dimer-dimer superposition along the sequences of the homodimer subunits of SARS-CoV-2 Nsp9 (black) and triSer-Nsp9 mutant (red), calculated from the snapshots selected every 1 ns from a 200 ns MD trajectory. The two subunits of each homodimer are indicated by separate traces. The green traces represent the average pairwise RMSD calculated upon dimer-dimer superposition of all mutant snapshots with all wild-type snapshots. The conclusion inferred from DOSY is consistent with the MD simulation results. The average pairwise RMSD values, upon mutant versus wild-type dimer-dimer superposition, are larger than the single species counterparts at both N-terminal and C-terminal tracts, implying that wild type and mutated Nsp9 fluctuate around different conformers. As those tracts are involved at the inter-monomer interface, [20] the pairwise RMSD profile is consistent with difference in the dimerization extent between the species.

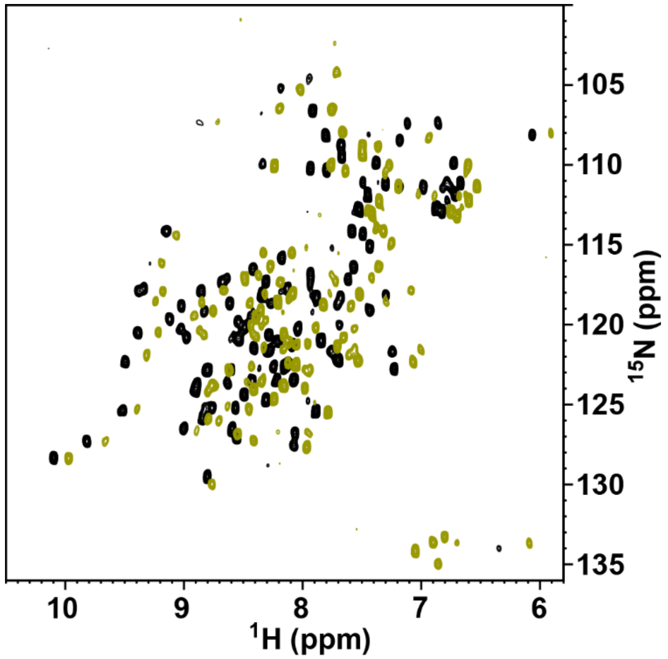

Figure S5. Overlay of the ^{15}N-^{1}H HSQC maps of SARS-CoV-2 Nsp9 at 298K (black contours) and 278 K (olive contours).

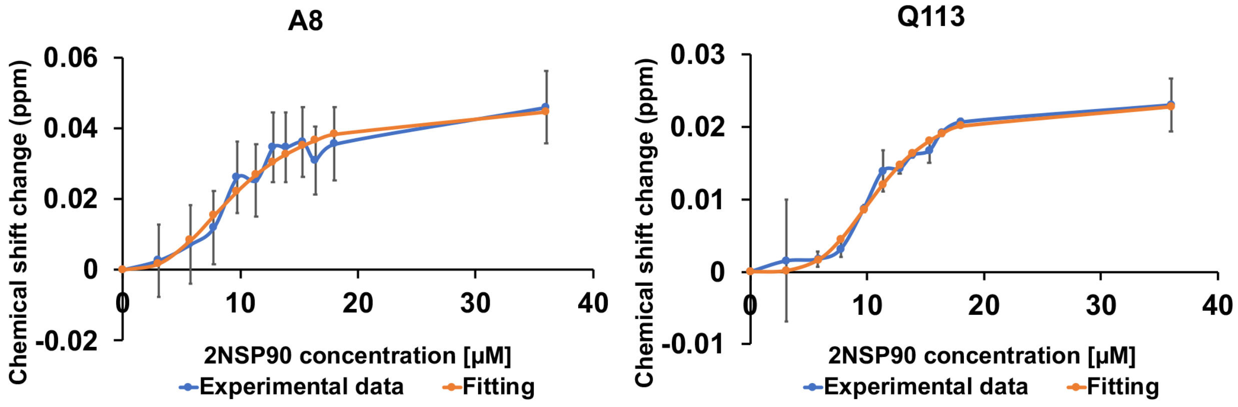

Figure S6. Fitting of the chemical shift changes observed at the peripheral residues of SARS-CoV-2 Nsp9 upon titration with 2NSP90. As reported in main text, besides the cross-peak loss observed with titrant increase, we could also detect progressive chemical shift changes. Those titration chemical shifts involve mostly the N-terminal and C-terminal residue signals, namely A8, L9, R111 and Q113 and a couple of other locations (C73, V76). This pattern is compatible with the intermediate exchange regime observed for all the other residues of Nsp9 and may arise for intrinsically mobile molecular locations where the chemical shift is effectively averaged by the local dynamics, leading to a very small difference between the limiting chemical shift values and matching therefore local fast exchange regime. The experimental data were fitted using a Hill-Langmuir model with equation:

$$\Delta\delta = \frac{\Delta\delta_{max} \times c^n}{K_A^n + c^n}$$

where $\Delta\delta$ and $\Delta\delta_{max}$ are the observed and maximum chemical shift change, *c* is the nanobody concentration, K_A is half occupation constant (related to the apparent dissociation constant) and *n* is the Hill coefficient. For A8, the fitting gave $n = 2.9 \pm 0.6$ (p value 1.5×10^{-3}) and $K_A = 9.7$ µM (p value 3.2×10^{-6}), whereas for Q113, $n = 4.0 \pm 0.5$ (p value 3.7×10^{-5}) and $K_A = 11$ µM (p value 4.1×10^{-9}). Albeit statistically significant, the fitting parameters should be regarded with some caution. This is expected due to difficulty of appreciating the limiting $\Delta\delta$ values and the experimental titration errors with concentrations as small as 18 µM (constant for Nsp9) and 36 µM (maximum for 2NSP90).

Table S1. The ELISA tests were performed on both Nsp9 dissolved in ammonium acetate (NSP) and dissolved in DMSO/PBS (INS). Uncoated blocked wells served as negative control (blank) for ELISA. Ratios of ELISA readouts between wells coated with Nsp9 and uncoated wells were taken as estimates of each nanobody's binding strength towards the antigen. Nanobodies exhibiting Strong INS/control or Nsp9/control ratios above 10 are considered strong binders.

Clone	CDR3 Group	ELISA INS	ELISA NSP	ELISA blank (control)	INS / control	NSP / control
2INS6	1	1.6606	2.0241	0.0969	17.13725	20.88854
2INS13	1	3.6286	3.8515	0.4403	8.241199	8.747445
2INS14	1	3.1991	2.4905	0.1328	24.08961	18.75377
2INS15	1	3.5749	3.5556	0.1695	21.09086	20.97699
2INS21	1	3.8533	3.7983	0.1839	20.95324	20.65416
2INS26	1	4.8694	4.3108	0.0816	59.67402	52.82843
2INS27	1	2.9974	2.7664	0.0957	31.32079	28.907
2INS30	1	4.2339	2.9781	0.1347	31.43207	22.10913
2INS31	1	2.8974	1.5437	0.1004	28.85857	15.3755
2INS32	1	4.4284	3.9266	0.1341	33.02312	29.28113
2INS34	1	4.358	3.6778	0.1321	32.99016	27.84103
2INS35	1	2.2232	2.0145	0.1006	22.0994	20.02485
2INS37	1	4.2254	3.4193	0.1418	29.79831	24.11354
2INS42	1	3.9214	2.2874	0.1279	30.65989	17.88428
2INS66	1	2.3038	1.377	0.1035	22.25894	13.30435
2INS76	1	4.309	3.8965	0.3283	13.12519	11.86872
2INS91	1	4.6538	3.3368	0.4607	10.10158	7.242891
3INS1	1	1.7013	1.3189	0.0934	18.2152	14.12099
3INS6	1	1.6434	1.3272	0.0863	19.04287	15.37891
3INS7	1	1.0084	0.7275	0.0897	11.24192	8.110368
3INS8	1	0.9664	0.4178	0.0849	11.3828	4.921084
3INS11	1	2.2977	2.5381	0.093	24.70645	27.2914
3INS16	1	2.8449	2.1031	0.1085	26.22028	19.38341
3INS21	1	3.9952	2.8413	0.2329	17.15414	12.19966

3INS26	1	3.3184	3.0078	0.1104	30.05797	27.24457
3INS32	1	3.0735	2.1801	0.1536	20.00977	14.19336
3INS35	1	2.5429	1.9874	0.0964	26.37863	20.61618
3INS37	1	1.3301	1.2324	0.0867	15.34141	14.21453
3INS42	1	2.3328	1.7834	0.1437	16.23382	12.41058
3INS49	1	2.5399	2.9157	0.1203	21.11305	24.23691
3INS50	1	2.0109	1.4152	0.0906	22.19536	15.62031
3INS67	1	2.547	1.9937	0.1076	23.671	18.52881
3INS72	1	2.5229	1.8334	0.1063	23.73377	17.24741
3INS74	1	1.5646	0.7418	0.0868	18.02535	8.546083
3INS86	1	1.322	1.6495	0.289	4.574394	5.707612
3INS92	1	2.0624	1.8056	0.1	20.624	18.056
2NSP1	2	2.5	4.5831	0.0901	27.74695	50.86681
2NSP9	2	2.989	5.6897	0.096	31.13542	59.26771
2NSP33	2	0.5101	1.6461	0.0857	5.952159	19.2077
2NSP38	2	3.1907	4.952	0.0897	35.57079	55.20624
2NSP55	2	3.3116	5.1433	0.0934	35.4561	55.06745
2NSP62	2	1.9995	3.5031	0.0781	25.60179	44.85403
2NSP79	2	2.87	5.32	0.0973	29.4964	54.67626
2NSP80	2	4.2058	5.3673	0.0973	43.22508	55.16238
2NSP92	2	2.91	4.9596	0.0905	32.1547	54.80221
3NSP4	2	2.5069	3.7444	0.0987	25.39919	37.93718
3NSP12	2	2.1605	3.5488	0.0892	24.22085	39.78475
3NSP28	2	0.6466	1.9015	0.0828	7.809179	22.96498
3NSP33	2	2.2006	3.4793	0.0848	25.95047	41.02948
3NSP52	2	2.004	2.9065	0.0849	23.60424	34.23439
3NSP58	2	1.5084	2.3769	0.0839	17.97855	28.33015
3NSP64	2	1.6567	2.1431	0.0876	18.9121	24.46461
3NSP65	2	4.3901	4.72	0.1698	25.85453	27.79741
3NSP66	2	2.0413	2.721	0.0845	24.1574	32.20118

3NSP69	2	1.8129	2.6048	0.0866	20.93418	30.07852
3NSP70	2	0.2614	0.5126	0.087	3.004598	5.891954
3NSP71	2	1.4571	2.0619	0.0876	16.63356	23.53767
3NSP79	2	1.9037	2.877	0.0822	23.15937	35
2NSP10	3	1.4958	4.4567	0.0847	17.65998	52.61747
2NSP15	3	0.7356	3.5612	0.0887	8.293123	40.14882
2NSP20	3	3.9084	5.1347	0.0946	41.31501	54.27801
2NSP21	3	2.3299	5.1093	0.3555	6.553868	14.37215
2NSP24	3	1.6673	4.3661	0.1074	15.52421	40.6527
2NSP47	3	0.226	0.5769	0.0929	2.432723	6.209903
2NSP71	3	0.2482	0.8837	0.0853	2.90973	10.35991
2NSP72	3	0.1514	0.6148	0.0867	1.746251	7.091119
2NSP82	3	1.4239	4.4291	0.0905	15.7337	48.94033
2NSP85	3	0.2501	1.2002	0.0904	2.766593	13.27655
2NSP95	3	2.782	4.784	0.112	24.83929	42.71429
3NSP63	3	0.3468	0.7881	0.0808	4.292079	9.753713
3NSP73	3	1.7099	2.4623	0.0851	20.09283	28.9342
3NSP90	3	2.1166	2.9898	0.0862	24.55452	34.68445
2NSP16	4	4.5913	5.5139	0.2076	22.11609	26.56021
2NSP27	4	4.0351	5.2543	0.1565	25.78339	33.5738
2NSP28	4	3.5767	5.2162	0.1294	27.64065	40.31066
2NSP30	4	2.4645	4.7373	0.1052	23.42681	45.03137
3NSP22	4	1.9257	3.8951	0.1072	17.96362	36.33489
3NSP25	4	2.1565	3.5611	0.1081	19.94912	32.94265
3NSP36	4	1.9548	2.8852	0.0891	21.93939	32.38159
3NSP86	4	1.6768	2.4579	0.1002	16.73453	24.52994
2NSP22	5	0.9622	4.1327	0.0947	10.16051	43.63992
2NSP48	5	1.0245	3.7108	0.0926	11.06371	40.07343
2NSP90	5	1.9764	4.7805	0.0941	21.00319	50.80234
3NSP11	5	1.941	3.3478	0.0887	21.88275	37.74295

3NSP38	5	0.6673	1.7741	0.0869	7.678941	20.41542
3NSP51	5	0.7785	1.8501	0.0948	8.212025	19.51582
3NSP76	5	1.2509	0.6085	0.0836	14.96292	7.278708
2INS47	6	6	6	0.8376	7.163324	7.163324
3INS38	6	2.8893	4.6169	0.1378	20.96734	33.50435
3INS76	6	3.1719	4.9016	0.4658	6.809575	10.52297
2NSP44	7	0.8601	2.2453	0.1242	6.925121	18.0781
2NSP83	7	3.1988	4.981	0.4271	7.489581	11.66237
3NSP47	7	0.538	1.3658	0.1193	4.50964	11.44845
2NSP23	8	1.7184	4.2818	0.1025	16.76488	41.77366
2NSP25	8	3.1576	5.2541	0.0889	35.51856	59.10124
2NSP70	8	1.5966	3.2321	0.0938	17.02132	34.45736
2INS10	9	5.1786	4.5834	0.1207	42.90472	37.97349
2INS46	9	6	5.4499	0.1133	52.95675	48.1015
2INS61	10	1.8398	5.3567	0.6683	2.752955	8.015412
3INS39	10	2.493	4.0747	0.922	2.703905	4.419414
3NSP49	11	0.7918	1.4154	0.0846	9.359338	16.7305
3NSP59	11	1.8859	2.7246	0.0851	22.16099	32.01645
2INS24	12	4.8489	1.4099	0.0965	50.24767	14.61036
2INS55	12	4.8303	0.539	0.0848	56.96108	6.356132
2INS43	13	0.4183	2.0533	0.119	3.515126	17.25462
3INS17	13	0.7256	2.7437	0.0913	7.947426	30.05148
2INS70	14	2.0679	5.0097	0.1236	16.73058	40.53155
2INS95	14	4.784	5.1837	0.1089	43.93021	47.60055
2INS58	15	2.8069	5.0288	0.0989	28.38119	50.84732
3INS93	15	1.0681	4.1258	0.0882	12.10998	46.77778
3NSP48	16	1.5108	2.7245	0.1937	7.79969	14.06557
3NSP81	16	0.6041	0.6598	0.0831	7.269555	7.939832
3NSP29	17	0.2019	0.4232	0.1099	1.837125	3.850773
2INS12	18	1.0694	3.5463	0.2595	4.121002	13.6659

2INS57	19	1.6643	0.7977	0.2502	6.651878	3.188249
2INS64	20	2.6154	4.431	0.0981	26.66055	45.1682
2INS44	21	4.2208	5.3692	0.4552	9.272408	11.79525
2INS45	22	2.8379	4.7531	0.1056	26.87405	45.01042
2INS71	23	0.6416	2.9364	0.149	4.30604	19.70738
2NSP40	24	1.0389	2.3909	0.111	9.359459	21.53964
3NSP39	25	0.2981	0.7045	0.0962	3.098753	7.323285
2INS39	26	3.4962	5.479	0.8981	3.892885	6.100657
3INS61	27	0.8272	2.8749	0.3178	2.602895	9.046256
3NSP35	28	0.4166	1.0508	0.0907	4.593164	11.58545
3NSP78	29	2.3553	3.8839	0.0823	28.61847	47.19198
2INS69	30	1.3515	1.3234	0.0958	14.10752	13.8142
2NSP11	31	1.3861	5.9479	0.0902	15.36696	65.94124
2NSP84	32	0.4116	1.2763	0.112	3.675	11.39554
2INS84	33	1.0445	2.2204	0.5491	1.902204	4.043708
2INS19	34	0.8157	0.4392	0.0902	9.043237	4.86918
2INS33	35	0.4922	0.2021	0.0699	7.041488	2.891273
3INS19	36	0.6433	0.2598	0.0909	7.077008	2.858086
3NSP31	37	3.2462	3.936	0.0878	36.97267	44.82916
3NSP56	38	0.2368	0.4968	0.0908	2.60793	5.471366
2INS48	39	1.9693	2.925	0.3374	5.836692	8.669235
2NSP32	40	0.6162	1.5921	0.089	6.923596	17.88876

Tables S2. Amino acid sequence analysis of the Nanobodies using the ProtParam tool allows the prediction of several theorical proprieties. MW, Molecular Weight; pI, isoelectric point; ε, extinction coefficient. Production yields of the Nsp9-specific nanobodies in the expression vector pHEN6 are also included.

Peak loss	Nanobody	CDR3 groups	Mw (Da)	pI	ε (0.1%) Cystines	ε (0.1%) Reduced	Yield
23270	2NSP23	8	14435.99	6.64	1.493	1.484	8.29
23294	2NSP90	5	14404.76	8.00	2.467	2.458	7.45
23314	3NSP52	2	14614.12	8.03	2.126	2.117	2.21
23327	3NSP78	29	14258.86	8.96	2.388	2.379	1.74
23342	2INS27	1	13414.86	8.64	1.607	1.597	4.24
23354	2INS45	22	13636.08	8.03	1.766	1.756	4.57
23362	2INS64	20	13166.73	8.69	1.828	1.819	14.05
23364	2INS69	30	14289.82	8.63	1.508	1.500	3.14

Table S3. Peak loss and attenuation in ^{15}N-^{1}H HSQC spectra of NSP9 titrations with nanobodies

**Peak loss**		
**Nanobody : NSP9 ratio**	**2NSP23[(a)]**	**2NSP90[(a)]**
0.17:1	-	-
0.32:1	-	*D50, W53*
0.43:1	*G17, F40, S46, K52, T67, L69, Y89*	*A28, L69*
0.54:1	*M12, C14, T18, T19, C23, A30, Y31, Y32, N33, T35, D50, W53, A54, F56, T64, Y66, Y87, L88, I91, G93, A108*	*M12, G17, T18, C23, A30, Y31, T35, F40, V41, L44, L45, K52, A54, F56, T67, E68, L88, Y89, G93, A108*
0.63:1	*Q11, S13, A15, A22, N27, L29, T34, G37, L44, L45, K58, D60, T62 I65, E68, E70, C73, K86, F90, N95*	*Q11, S13, A15, T19, T21, A22, T24, N27, L29, Y32, N33, N33sc1, N33sc2 T34, G37, G38, S46, W53sc, T62, G63, T64, Y66, E70, R74, I91, N95*

**Fast peak attenuation [(b)]**	
**2NSP23**	**2NSP90**
C14, G17, T18, T19, A30, Y32, F40, S46, K52, A54, F56, T67, L69, Y89, G93, A108	*S13, A30, Y31, N33, V41, A43, L51, W53, A54, F56, T67, L69, A108, R111*

(a) Although quite similar in the relative interactions, the two nanobodies exhibit slight differences for the involved epitopes, with 2NSP23 addressing first the surface encompassing the fragments 50-53, 86-89, and 2NSP90 selecting only fragment 50-53.
(b) The peaks with the steepest decrease in intensity were identified when the slope of their relative intensity attenuations was larger than the average attenuation slope increased by one standard deviation.
